# Molecular characterization of amikacin, kanamycin and capreomycin resistance in M/XDR-TB strains isolated in Thailand

**DOI:** 10.1186/1471-2180-14-165

**Published:** 2014-06-22

**Authors:** Angkanang Sowajassatakul, Therdsak Prammananan, Angkana Chaiprasert, Saranya Phunpruch

**Affiliations:** 1 Department of Biology, Faculty of Science, King Mongkut’s Institute of Technology Ladkrabang, Bangkok 10520, Thailand; 2 Tuberculosis Research Laboratory, National Center for Genetic Engineering and Biotechnology, National Science and Technology Development Agency, Thailand Science Park, Pathumthani 12120, Thailand; 3 Department of Microbiology, Faculty of Medicine Siriraj Hospital, Mahidol University, Bangkok 10700, Thailand; 4 Drug Resistance Tuberculosis Research Fund, Siriraj Foundation, Bangkok 10700, Thailand

**Keywords:** Tuberculosis, Second-line drug, Resistance, Aminoglycoside

## Abstract

**Background:**

The emergence of multidrug-resistant tuberculosis (MDR-TB) and extensively drug-resistant tuberculosis (XDR-TB) makes the treatment and control of tuberculosis difficult. Rapid detection of drug-resistant strains is important for the successful treatment of drug-resistant tuberculosis; however, not all resistance mechanisms to the injectable second-line drugs such as amikacin (AK), kanamycin (KM), and capreomycin (CAP) are well understood. This study aims to validate the mechanisms associated with AK, KM, and CAP resistance in *M. tuberculosis* clinical strains isolated in Thailand.

**Results:**

A total of 15,124 *M. tuberculosis* clinical strains were isolated from 23,693 smear-positive sputum samples sent from 288 hospitals in 46 of 77 provinces of Thailand. Phenotypic analysis identified 1,294 strains as MDR-TB and second-line drugs susceptibility was performed in all MDR-TB strains and revealed 58 XDR-TB strains. Twenty-nine KM-resistant strains (26 XDR-TB and 3 MDR-TB) could be retrieved and their genes associated with AK, KM, and CAP resistance were investigated compared with 27 KM-susceptible strains. Mutation of the *rrs* (A1401G) was found in 21 out of 29 KM-resistant strains whereas mutations of *eis* either at C-14 T or at G-37 T were found in 5 strains. Three remaining KM-resistant strains did not contain any known mutations. Capreomycin resistance was determined in 28 of 29 KM-resistant strains. Analysis of *tlyA* revealed that the A33G mutation was found in all CAP-resistant strains and also in susceptible strains. In contrast, the recently identified *tlyA* mutation T539G and the novel Ins49GC were found in two and one CAP-resistant strains, respectively. In addition, our finding demonstrated the insertion of cytosine at position 581 of the *tap*, a putative drug efflux encoding gene, in both KM-resistant and KM-susceptible strains.

**Conclusions:**

Our finding demonstrated that the majority of KM resistance mechanism in Thai *M. tuberculosis* clinical strains was *rrs* mutation at A1401G. Mutations of the *eis* promoter region either at C-14 T or G-37 T was found in 5 of 29 strains whereas three strains did not contain any known mutations. For CAP resistance, 3 of 28 CAP-resistant strains contained either T539G or Ins49GC mutations at *tlyA* that might be associated with the resistant phenotype.

## Background

Tuberculosis (TB) is a global public health problem caused by an infection with *Mycobacterium tuberculosis*. There were approximately 9 million new cases of TB and 1.3 million deaths in 2012 [1]. The emergence of multidrug-resistant TB (MDR-TB; resistance at least to isoniazid and rifampicin) and extensively drug-resistant TB (XDR-TB; MDR-TB plus resistance to any fluoroquinolones and one of the second-line injectable drugs, amikacin, kanamycin and capreomycin) remains a global health problem that hinders the prevention, treatment, and control of TB. In Thailand, approximately 80,000 new TB cases were notified in 2012 and MDR-TB appeared in 1.7% and 35% of new TB cases and previously treated TB cases, respectively [1].

Rapid identification of drug-resistant strains is one of the major strategies for fighting against TB. Molecular-based methods for detection of drug resistance genes have been shown to be a promising method for identification of drug-resistant strains; for example, the Xpert MTB/RIF assay and the GenoType MTBDRplus assay have been successfully used to identify rifampicin-resistant *M. tuberculosis* and MDR-TB, respectively [2-7]. In contrast, knowledge concerning resistance mechanisms of the second-line anti-TB drugs is still limited. Better understanding of the resistance mechanisms of these drugs could lead to the development of a high sensitive test for detection of the resistance genes and also promote the use of molecular-based methods for screening the strains resistant to second-line drugs, including the XDR-TB strain.

The aminoglycosides amikacin (AK) and kanamycin (KM) are the second-line injectable drugs used to treat MDR-TB. The drugs bind to 16S rRNA in the 30S small ribosomal subunit and inhibit protein synthesis [8]. Mutations in the *rrs* gene encoding 16S rRNA are associated with high-level drug resistance in *M. tuberculosis*; the *rrs* A1401G mutation is the most frequently reported mutation and has been identified in 30 to 90% of KM-resistant *M. tuberculosis* strains [9-12].

Recently, overexpression of the aminoglycoside acetyltransferase-encoding gene, *eis*, has been associated with a low-level resistance to KM [13,14]. This overexpression resulted from either point mutations in the promoter region of the *eis* gene or mutations in the 5′ untranslated region (UTR) of the *whiB7* gene, which encodes a putative regulator of the *eis* gene. This type of *eis* promoter mutation was found in 26-80% of KM-resistant *M. tuberculosis* clinical strains [14-17]. However, some resistant strains do not contain any known mutations. Other possible resistance mechanisms, including the presence of drug efflux pumps or enzymes that can inactivate the drug or modify the drug target, have been proposed. Tap, a putative efflux pump that was originally described in *Mycobacterium fortuitum*, conferred resistance to tetracycline and aminoglycosides when introduced into *M. smegmatis*[18]. A homolog of this protein (Rv1258c) has been found in *M. tuberculosis* and functions under the control of WhiB7 [19].

Previous studies demonstrated that the *rrs* mutation conferring KM resistance also exhibited the cross-resistance to capreomycin (CAP), a cyclic polypeptide antibiotic [20,21]. Capreomycin binds across the 23S rRNA helix 69 and 16S rRNA helix 44 of the ribosome, resulting in inhibiting the protein synthesis [22,23]. Resistance to CAP has been reported to correlate with the gene encoding 2´-O-methyltransferase (*tlyA*) [24], although it is not a sensitive genetic marker for CAP resistance due to the infrequent finding [16]. TlyA functions by methylating at nucleotide C1409 in helix 44 of 16S rRNA and nucleotide C1920 in helix 69 of 23S rRNA. Loss of this methylation confers resistance to CAP and viomycin [23].

The present study aimed to validate all reported mechanisms associated with AK, KM and CAP resistance in M/XDR-TB clinical strains isolated in Thailand. Moreover, these mechanisms were also investigated in KM–susceptible strains.

## Results

### Amikacin- and kanamycin-resistant phenotypes

A total of 15,124 *M. tuberculosis* clinical strains were isolated from 23,693 smear-positive sputum samples sent from 288 hospitals in 46 of 77 provinces of Thailand. Phenotypic analysis identified 1,294 strains as MDR-TB. Using the standard proportion method on M7H10 agar with a single concentration of 1 μg/ml for ofloxacin and 6 μg/ml for AK and KM, 58 strains were defined as XDR-TB. Twenty-nine KM-resistant strains (26 XDR-TB and 3 MDR-TB) could be retrieved and available for further investigation on the genes associated with AK, KM, and CAP resistance (Additional file 1: Table S1). MICs of AM, KM, and CAP were determined, and the results are summarized in Table 1.

**Table 1:** **Genetic characterization of genes associated with KM resistance of KM-resistant and KM-susceptible *M. tuberculosis *** **strains**

**No. of strains**	**MIC (μg/ml)**			**Gene/Mutation**				
	**AK**	**KM**	**CAP**	***rrs***	***eis***	***tap***	***whiB7***	***tlyA***
KM resistant (29)								
1	>64	>64	>64	A1401G	wt	Ins581C	wt	A33G^b^
7	>64	>64	32	A1401G	wt	Ins581C	wt	A33G^b^
5	>64	>64	32	A1401G	wt	wt	wt	A33G^b^
4^a^	>64	>64	16	A1401G	wt	Ins581C	wt	A33G^b^
2	>64	>64	16	A1401G	wt	wt	wt	A33G^b^
1	>64	>64	4	A1401G	wt	Ins581C	wt	A33G^b^
1	8	32	8	A1401G	wt	Ins581C	wt	A33G^b^
1	8	>64	8	wt	C-14 T	Ins581C	wt	A33G^b^
1	8	>64	>64	wt	C-14 T	Ins581C	wt	A33G^b^/Ins49GC
2^a^	8	>64	>64	wt	C-14 T	Ins581C	wt	A33G^b^/T539G
1	8	>64	>64	wt	G-37 T	Ins581C	wt	A33G^b^
2	>64	>64	16	wt	wt	Ins581C	wt	A33G^b^
1^a^	>64	>64	16	wt	wt	wt	wt	A33G^b^
KM susceptible (27)								
5	2-4	4	2-4	wt	wt	Ins581C	wt	A33G^b^
22	2-4	4	2-4	wt	wt	wt	wt	A33G^b^

^a^include one MDR-TB strain; ^b^no amino acid change.

### Molecular analysis of genes associated with amikacin, kanamycin, and capreomycin resistance

The 16S rRNA genes (*rrs*) of all 29 KM-resistant strains were amplified and sequenced. The results revealed a point mutation at nucleotide position 1401 (A → G), which corresponds to position 1408 of the *Escherichia coli rrs* gene, in 21 strains (Table 1). Almost all strains harboring the *rrs* A1401G mutation showed a high-level of resistance to both AK and KM, with MICs >64 μg/ml, whereas variable MICs were found against CAP, with ranging from 4 to >64 μg/ml (Table 1).

The nucleotide sequences of coding regions and the putative promoter regions of *eis* (Rv2416c) and *whiB7* (Rv3197A), coding regions of *tap* (Rv1258c) and *tlyA* (Rv1694), were investigated in all KM-resistant clinical strains and 27 KM-susceptible clinical strains. No mutation of all investigated genes (except for *tap*) was found in 21 strains with *rrs* mutation. For the remaining eight KM-resistant strains, point mutations at either position -14 (C → T) or position -37 (G → T) upstream of the *eis* gene were observed in 5 strains; the C-14 T mutation was found in 4 strains, whereas the G-37 T mutation was found in only one strain (Table 1 and Additional file 1: Table S1). No *eis* mutations were found in 27 KM-susceptible strains (Table 1 and Additional file 2: Table S2). Sequence analysis of the *whiB7* gene and its promoter region did not reveal any mutations in all KM-resistant and -susceptible strains (Table 1).

Investigation of the *tap* gene in KM-resistant strains revealed that almost all strains (except one strain) with Beijing genotype exhibited the insertion of cytosine between position 580 and 581 of the *tap* gene (Additional file 1: Table S1). This insertion caused a frameshift mutation and a premature stop codon, resulting in the production of a truncated protein (reduced in size from 419 to 231 amino acids). However, analysis of KM-susceptible strains also revealed this mutation (5 out of 27 strains) (Table 1 and Additional file 2: Table S2).

Sequence analysis of the *tlyA* gene revealed A → G nucleotide substitution at position 33 in all KM-resistant strains; however this mutation did not confer any amino acid change (Table 1 and Additional file 1: Table S1). Two CAP-resistant strains showed the T → G nucleotide substitution at position 539 of *tlyA* that caused the amino acid change from lysine to arginine (L → R) at codon 180 (Additional file 1: Table S1). One strain showed an insertion of GC at position 49, resulting in a frameshift mutation and the reduction of amino acid size from 268 to 26 amino acids (Additional file 1: Table S1). However, the A33G mutation, but not other *tlyA* mutations, was also found in all susceptible strains (Table 1 and Additional file 2: Table S2).

## Discussion

In this study, the genetic mutations associated with resistance to AK, KM, and CAP were investigated in 26 XDR- and 3 MDR-TB strains isolated in Thailand. A nucleotide substitution from A to G at position 1401 (corresponding to position 1408 of the *E. coli rrs* gene) of the *rrs* gene is the most common mutation conferring high-level resistance to AK and KM in *M. tuberculosis*. Although approximately 30-90% of resistant strains contain this mutation [9-12], other mutations, including C1402T and G1484T, have also been reported [25-29]. The A1401G mutation has been preferentially used as a surrogate marker for resistance to AK and KM, whereas other *rrs* mutations are poor markers due to their presence in susceptible strains [30]. Our results revealed that the A1401G mutation was present in 21 of 29 KM-resistant clinical strains, and no other *rrs* mutations were identified (Table 1). Almost all of these strains (20 out of 21) had MICs >64 μg/ml for both AK and KM while they showed broad MICs ranging from 4 to 64 μg/ml for CAP. This is consistent with previous studies reporting that the *rrs* A1401G mutation is the most common mechanism of KM resistance and correlates with high-level resistance [21,31,32]. In addition, this mutation also confers cross-resistance to CAP [31]. The eight KM-resistant strains lacking the *rrs* mutation showed high-level resistance to KM (MIC >64 μg/ml), but five of them had a lower MIC for AK (MIC of 8 μg/ml), indicating that other resistance determinants are involved in their resistance phenotype.

Investigation of other reported resistance mechanisms revealed that five of them had mutations in the promoter region of the *eis* gene, which encodes an aminoglycoside acetyltransferase (Table 1). This aminoglycoside acetyltransferase (Eis) catalyzes the transfer of an acetyl group from acetyl-coenzyme A to an amine group of aminoglycoside. It has been reported that Eis of *M. tuberculosis* shows a multiacetylation capability at the 2′-, 3- or 6′ positions of aminoglycoside antibiotics, resulting in an inactivation of many aminoglycoside antibiotics, including neamine, hygromycin, kanamycin, and amikacin [33]. In this study, all five strains harboring *eis* promoter mutations showed high-level KM resistance but low-level resistance to AK. The most identified mutation was C-14 T (4 of 5 strains). These mutations and other *eis* mutations, such as G-6 T, G-10A, C-12 T, A-13G and C-15 T, have been previously shown to be associated with KM resistance [14,16,17]. Zaunbrecher et al. (2009) have reported that the major *eis* promoter mutations were G-10A and C-14 T [14]. Overexpression of *eis* resulting from the C-14 T mutation caused the highest levels of *eis* transcript, followed by G-37 T, G-10A, C-12 T and A-13G mutations [14]. In contrast to the previous study indicating that overexpression of *eis* confers low-level resistance to KM [14], our results revealed that the strains harboring *eis* mutation expressed high-level resistance to KM. One possible explanation is that these strains have additional unknown mechanisms contributing to their KM resistance, and these generate high-level resistance in combination with the *eis* mutation.

Other resistance determinants that are thought to be involved in resistance to AK, KM, and other structurally unrelated aminoglycosides (i.e., streptomycin) were also investigated in this study. The Tap protein is a putative multidrug efflux pump that was originally described in *M. fortuitum*[18]. Rv1258c encodes the homologous Tap protein in *M. tuberculosis*. Introduction of the *tap* gene from *M. fortuitum* into *M. smegmatis* conferred low-level resistance to tetracycline and aminoglycosides [18,34,35]. Our results revealed an insertion of cytosine between positions 580 and 581 of *tap* in 21 of 29 KM-resistant strains. This mutation leads to a frameshift mutation at codon 194 resulting in the production of a truncated protein, reduced in size from 419 to 231 amino acids, that is likely to affect Tap activity. However, this insertion was also found in KM-susceptible clinical strains, suggesting that this protein is not associated with AK and KM resistance in *M. tuberculosis*. Interestingly, all of these *tap* mutation was found in the Beijing strains. This result was consistent with recent studies demonstrated that this type of mutation was found in all *M. tuberculosis* Beijing strains isolated from Russia, South Africa, the United Kingdom, and Spain [36,37] and confirmed the observation that an insertion of cytosine between positions 580 and 581 of *tap* is a polymorphism specific to the Beijing family of *M. tuberculosis*[37].

An association of WhiB7, a transcriptional regulator, with the expression of at least two antibiotic resistance genes, *eis* and *tap* has been demonstrated [19]. An increase in *whiB7* expression, resulting from mutations located in the 5′ untranslated region (UTR), leads to upregulation of *eis* and *tap,* conferring low-level resistance to KM and streptomycin, respectively [13]. Investigation of this gene and its 5′ UTR revealed no mutations in any KM-resistant and -susceptible strains. However, its expression level was not determined in this study.

Previous report revealed that lack of 2′-O-methyltranferase, which is encoded by *tlyA* and functions by methylation of specific nucleotides in 16S rRNA and 23S rRNA, resulted in CAP resistance [23]. Investigation of the *tlyA* showed that all tested strains had the A33G substitution without any amino acid changes, suggesting that this mutation is only nucleotide polymorphism and not associated with the resistant phenotype. Other *tlyA* mutations, T539G and Ins49GC, were found in two and one CAP-resistant strains, respectively, but were not found in all CAP-susceptible strains. These strains exhibited the high-level resistance to CAP with MIC greater than 64 μg/ml and did not contain the *rrs* mutation, indicating that these mutations were expectedly associated with CAP resistance [24]. Most recently, the T539G has been reported in capreomycin-resistant isolates in Korea but with low percentage (3 out of 86, 3.5%) [38].

## Conclusions

The most frequent AK- and KM-resistant mechanism in *M. tuberculosis* clinical strains isolated in Thailand was the *rrs* A1401G mutation (21 of 29 strains). This mutation correlated with high-level resistance to both AK and KM, and also showed cross-resistance to CAP. Mutations of the *eis* promoter region are associated with low-level resistance to AK and found in 5 out of 29 KM-resistant strains. Two types of mutations at *tlyA* were found in this study and might be associated with CAP resistance. Identification of the resistant mechanisms, particularly a novel mechanism, is important for the development of surrogate markers that can be combined with other known resistance determinants to improve the rapid detection of drug-resistant *M. tuberculosis* strains.

## Methods

### Mycobacterial strains and culture conditions

*Mycobacterium tuberculosis* clinical strains (one strain per patient) were obtained from the Drug-Resistant Tuberculosis Research Laboratory, Drug-Resistant Tuberculosis Research Fund, Siriraj Foundation, Faculty of Medicine Siriraj Hospital, Mahidol University. They were isolated between 2004 and 2011 from new and previously treated patients with both known and unknown HIV status. This study was approved by the Siriraj Ethics Committee, Mahidol University, Bangkok, Thailand (Certificate of Approval No. Si 208/2005). The mycobacteria were cultured on Löwenstein-Jensen (LJ) medium (BBL, USA) and incubated at 37°C for 3-4 weeks. Species identification and antimycobacterial susceptibility testing were performed using in-house one-tube multiplex PCR [39] and the standard proportion method [40,41], respectively.

### Isolation of genomic DNA

One loop of mycobacterial cells grown on solid medium was scraped and suspended in 500 μl of TE buffer (10 mM Tris-HCl (pH8.0), 1 mM EDTA). The cells were inactivated by heating at 80°C for 20 min and subsequently harvested by centrifugation at 6,000xg at 4°C for 10 min. The cells were resuspended in 400 μl of Tris-EDTA-Tween-lysozyme solution (10 mM Tris-HCl (pH 8.0), 1 mM EDTA, 0.5% (v/v) Tween 80, 2 mg/ml lysozyme (Amresco, USA)), and the mixtures were then incubated at 37°C for 3 h. SDS and proteinase K were added to the cell suspension to generate final concentrations of 1% (w/v) and 1 mg/ml, respectively, prior to incubation at 37°C for 1 h. Then, 80 μl of 5 M NaCl and 80 μl of 10% (w/v) cetyl trimethyl ammonium bromide (CTAB) (Sigma, USA) were added to the suspension, and the suspension was immediately heated at 65°C for 15 min. An equal volume of chloroform-isoamyl alcohol (24:1) (v/v) was added to the suspension. The aqueous DNA phase was separated by centrifugation at 12,000xg for 5 min and mixed again with an equal volume of chloroform-isoamyl alcohol (24:1) (v/v). DNA was precipitated by adding 0.1 volume of 3 M sodium acetate (pH 5.3) and 2.5 volumes of ice-chilled absolute ethanol, followed by incubation at -70°C for 30 min. DNA was separated by centrifugation at 12,000xg at 4°C for 15 min. Total nucleic acid was washed once with 500 μl of ice-chilled 70% ethanol, dried, and resuspended in 20 μl of TE buffer. RNaseA (Qiagen, Germany) was added to the total nucleic acid solution to generate a final concentration of 0.5 μg/μl, and the tube was subsequently incubated at 37°C for 1 h.

### DNA amplification

The *rrs* (Rvnr01), *eis* (Rv2416c), *whiB7* (Rv3197A), *tap* (Rv1258c), and *tlyA* (Rv1694) genes were amplified by PCR using specific primers (Table 2). PCR was performed in a 50-μl reaction mixture containing 20 mM Tris-HCl (pH 8.4), 50 mM KCl, 1.5 mM MgCl_2_, 200 μM of each dNTP, 0.5 μM of each primer, 50 ng of DNA template, and 2.5 U of *Taq* DNA polymerase (Promega, USA). The PCR conditions consisted of an initial denaturation at 94°C for 5 min, followed by 35 cycles of denaturation at 94°C for 30 sec, annealing at 56-60°C for 1 min and extension at 72°C for 1-2 min depending on the PCR product size (Table 2), and a final extension at 72°C for 7 min. The PCR products were analyzed by agarose gel electrophoresis and purified using the QIAquick PCR Purification Kit (Qiagen, Germany) prior to submission for DNA sequencing.

**Table 2:** **Primers used for amplification and sequencing of *M.tuberculosis *** **clinical strains**

**Gene**	Primer name (position*)	**Primer sequence (5′→3′)**	**Annealing temp (°C)**	**PCR product size (bp)**	**Purpose**	**Reference**
*rrs*	F-rrs (-44)	5′-TTCTAAATACCTTTGGCTCCCT-3′	51	1,680	PCR/Seq	[42]
	R-rrs (1,636)	5′-TGGCCAACTTTGTTGTCATGCA-3′	53		PCR/Seq	[42]
	F-rrs1 (554)	5′-CTGGGCGTAAAGAGCTCGTA-3′	54		Seq	This study
	F-rrs2 (1,114)	5′-GTTGCCAGCACGTAATGGTG-3′	54		Seq	This study
	R-rrs1 (483)	5′-TCCACCTACCGTCAATCCGA-3′	54		Seq	This study
	R-rrs2 (1,073)	5′-ATCTCACGACACGAGCTGAC-3′	54		Seq	This study
*eis* (Rv2416c)	F-Rv2417c (-316)	5′-GCGGTGCATCACGTCGCCGA-3′	60	1,661	PCR/Seq	This study
	R-eis-Rv2415c (1,345)	5′-GCAACGCGATCCGCGAGTGC-3′	60		PCR/Seq	This study
	F-eis1 (247)	5′-AGTTTCGTCGCGGTGGCGCC-3′	60		Seq	This study
	F-eis2 (816)	5′-GGACCCGTTACCCCACCTGC-3′	60		Seq	This study
	R-eis1 (240)	5′-GGCGGTCGGGAGCACCACTT-3′	60		Seq	This study
	R-eis2 (769)	5′-TCAGGGCCCGCCACAACGCA-3′	60		Seq	This study
*tap* (Rv1258c)	F-Rv1259 (-496)	5′-CAGGCCGGCCCTATGCAGTG-3′	60	1,847	PCR/Seq	This study
	R-Rv1257c (1,351)	5′-CGGTCTTGCCGGTAGCCGTC-3′	60		PCR/Seq	This study
	F-tap1 (41)	5′-TCGCAACGCTGATGGCGGCC-3′	60		Seq	This study
	F-tap2 (641)	5′-AGGGGCTGCGCTTCGTCTGG-3′	60		Seq	This study
	R-tap1 (210)	5′-CCCGAAGTAGTCGACCGCGG-3′	60		Seq	This study
	R-tap2 (862)	5′-GACGGGGAACGCGGATAGCC-3′	60		Seq	This study
*whiB7* (Rv3197A)	F URT-whiB7 (-451)	5′-GCTGGTTCGCGGTCGGACCT-3′	60	550	PCR/Seq	This study
	R whiB7 (99)	5′-CGGGGTATCGGCGAACCACA-3′	58		PCR/Seq	This study
*tlyA* (Rv1694)	F-tlyA (1)	5′-GTGGCACGACGTGCCCGCGT-3′	62	807	PCR/Seq	This study
	R-tlyA (807)	5′-CTACGGGCCCTCGCTAATCG-3′	58		PCR/Seq	This study

*The first 5′nucleotide position of each primer was counted from the translation start codon of each gene.

### DNA sequencing analysis

Nucleotide sequencing was performed with the Big-Dye™ Terminator Cycle Sequencing Ready Reaction Kit (Perkin Elmer, USA) using an ABI PRISM^R^ 3700 DNA analyzer at First BASE Laboratories (Malaysia). The PCR products were sequenced in both directions. The obtained nucleotide sequences were compared with those of *M. tuberculosis* H37Rv (Accession no. NC_000962) by pairwise alignment using the ClustalW program [43].

### Drug susceptibility testing and determination of minimal inhibitory concentrations (MICs)

The susceptibility testing was achieved by the disc diffusion method on the Middlebrook 7H10 agar (Difco, USA) supplemented with 10% oleic acid-albumin-dextrose-catalase (OADC) with a single concentration of drug as recommended by the CLSI [41]. The MICs of AM, KM, and CAP were determined by the agar dilution method according to CLSI guidelines [41] on Middlebrook 7H10 agar supplemented with 10% OADC and various concentrations of drug (0, 2, 4, 8, 16, 32, and 64 μg/ml). AK, KM, and CAP were purchased from Sigma Aldrich (Germany). The MIC was defined as the lowest concentration of drug that inhibited growth (>99%) after 4 weeks of incubation at 37°C. *M. tuberculosis* H37Rv ATCC 27294 was used as the susceptible control strain. Three independent experiments were performed for each strain.

## Competing interests

The authors declare that they have no competing interests.

## Authors’ contributions

AS performed all experiments in this study and drafted the manuscript. AS, TP, and SP analyzed the results and formatted the data. TP and SP conceptualized and designed the experimental procedures, supervised all the experimental works, corrected and produced the final version of the manuscript. AC provided clinical MTB strains from Thai patients. SP provided funding and grant. All authors read and approved the final manuscript.

## Supplementary Material

Additional file 1: Table S1 Genetic characterization of resistance genes and MIC values for amikacin, kanamycin and capreomycin in 29 KM-resistant clinical isolates of *M. tuberculosis.* Click here for file

Additional file 2: Table S2 Genetic characterization of resistance genes and MIC values for amikacin, kanamycin and capreomycin in 27 AK- and KM-susceptible clinical isolates of *M. tuberculosis.* Click here for file
