# Supplementary material for: Molecular characterization of amikacin, kanamycin and capreomycin resistance in M/XDR-TB strains isolated in Thailand
Source: BMC Microbiol. 2014 Jun 22;14:165. doi: 10.1186/1471-2180-14-165 (PMC4076439; doi:10.1186/1471-2180-14-165)
Supplement: Additional file 1: Table S1 — Genetic characterization of resistance genes and MIC values for amikacin, kanamycin and capreomycin in 29 KM-resistant clinical isolates of M. tuberculosis. [file 1471-2180-14-165-S1.doc]

**Additional file 1: Table S1** Genetic characterization of resistance genes and MIC values for amikacin, kanamycin and capreomycin in 29 KM-resistant clinical isolates of *M. tuberculosis*

| **Isolate** | **Drug resistance** | **Genotype** | **MIC (µg/ml)** | | |  | ***rrs* mutation** |  | ***eis* promoter mutation** | ***tap* mutation** | | ***whiB7* promoter mutation** | ***tlyA* mutation** | |
| --- | --- | --- | --- | --- | --- | --- | --- | --- | --- | --- | --- | --- | --- | --- |
|  |  |  | **AK** | **KM** | **CAP** |  |  |  |  | **Nucleotide change** | **Amino acid change** |  | **Nucleotide change** | **Amino acid change** |
| MT012 | XDR | NBJ | >64 | >64 | 16 |  | A1401G |  | wt | wt | wt | wt | A33G | wt |
| MT016 | XDR | BJ | >64 | >64 | 16 |  | A1401G |  | wt | Ins581C | Frameshift (419>231) | wt | A33G | wt |
| MT058 | XDR | BJ | >64 | >64 | 16 |  | A1401G |  | wt | Ins581C | Frameshift (419>231) | wt | A33G | wt |
| MT092 | XDR | BJ | >64 | >64 | 4 |  | A1401G |  | wt | Ins581C | Frameshift (419>231) | wt | A33G | wt |
| MT104 | XDR | BJ | >64 | >64 | 32 |  | A1401G |  | wt | Ins581C | Frameshift (419>231) | wt | A33G | wt |
| MT109 | XDR | BJ | >64 | >64 | >64 |  | A1401G |  | wt | Ins581C | Frameshift (419>231) | wt | A33G | wt |
| MT111 | XDR | NBJ | >64 | >64 | 32 |  | A1401G |  | wt | wt | wt | wt | A33G | wt |
| MT120 | XDR | BJ | 8 | >64 | >64 |  | wt |  | C-14T | Ins581C | Frameshift (419>231) | wt | A33G  Ins49GC | wt  Frameshift (268>26) |
| MT127 | XDR | NBJ | >64 | >64 | 32 |  | A1401G |  | wt | wt | wt | wt | A33G | wt |
| MT164 | XDR | NA | >64 | >64 | 16 |  | wt |  | wt | Ins581C | Frameshift (419>231) | wt | A33G | wt |
| MT179 | XDR | BJ | >64 | >64 | 32 |  | A1401G |  | wt | Ins581C | Frameshift (419>231) | wt | A33G | wt |
| MT182 | XDR | NBJ | >64 | >64 | 16 |  | A1401G |  | wt | wt | wt | wt | A33G | wt |
| MT260 | XDR | BJ | 8 | >64 | >64 |  | wt |  | C-14T | Ins581C | Frameshift (419>231) | wt | A33G  T539G | wt  L180R |
| MT287 | XDR | NBJ | >64 | >64 | 32 |  | A1401G |  | wt | wt | wt | wt | A33G | wt |
| MT381 | XDR | BJ | >64 | >64 | 32 |  | A1401G |  | wt | Ins581C | Frameshift (419>231) | wt | A33G | wt |
| MT388 | XDR | BJ | >64 | >64 | 16 |  | A1401G |  | wt | Ins581C | Frameshift (419>231) | wt | A33G | wt |
| MT515 | XDR | NBJ | >64 | >64 | 32 |  | A1401G |  | wt | wt | wt | wt | A33G | wt |
| MT592 | XDR | BJ | >64 | >64 | 32 |  | A1401G |  | wt | Ins581C | Frameshift (419>231) | wt | A33G | wt |
| MT653 | XDR | BJ | >64 | >64 | 32 |  | A1401G |  | wt | Ins581C | Frameshift (419>231) | wt | A33G | wt |
| MT688 | XDR | BJ | 8 | 32 | 8 |  | A1401G |  | wt | Ins581C | Frameshift (419>231) | wt | A33G | wt |
| MT810 | XDR | BJ | >64 | >64 | 32 |  | A1401G |  | wt | Ins581C | Frameshift (419>231) | wt | A33G | wt |
| MT825 | XDR | BJ | >64 | >64 | 16 |  | wt |  | wt | Ins581C | Frameshift (419>231) | wt | A33G | wt |
| MT848 | XDR | NBJ | >64 | >64 | 32 |  | A1401G |  | wt | wt | wt | wt | A33G | wt |
| MT962 | XDR | BJ | >64 | >64 | 32 |  | A1401G |  | wt | Ins581C | Frameshift (419>231) | wt | A33G | wt |
| MT966 | XDR | BJ | 8 | >64 | >64 |  | wt |  | G-37T | Ins581C | Frameshift (419>231) | wt | A33G | wt |
| MT984 | XDR | BJ | 8 | >64 | 8 |  | wt |  | C-14T | Ins581C | Frameshift (419>231) | wt | A33G | wt |
| MT433 | MDR | BJ | >64 | >64 | 16 |  | wt |  | wt | wt | wt | wt | A33G | wt |
| MT617 | MDR | BJ | 8 | >64 | >64 |  | wt |  | C-14T | Ins581C | Frameshift (419>231) | wt | A33G  T539G | wt  L180R |
| MT845 | MDR | BJ | >64 | >64 | 16 |  | A1401G |  | wt | Ins581C | Frameshift (419>231) | wt | A33G | wt |

wt, wild-type; MDR, Multidrug resistance; XDR, Extensively drug resistance; BJ, Beijing; NBJ, Non-Beijing; C-14T, Nucleotide change at position -14 from cytosine to thymine; G-37T, Nucleotide change at position -37 from guanine to thymine; Ins581C, Insertion of cytosine at position 581; A33G, Nucleotide change at position 33 from adenine to guanine; Ins49GC, Nucleotide change at position 49 by insertion of guanine and cytosine; T539G, Nucleotide change at position 539 from thymine to guanine; L180R, Amino acid change at codon 180 from leucine to arginine; Frameshift (419>231) and frameshift (268>26), Mutation caused size reduction from 419 or 268 to 231 or 26 residues, respectively
