# Supplementary material for: Molecular characterization of amikacin, kanamycin and capreomycin resistance in M/XDR-TB strains isolated in Thailand
Source: BMC Microbiol. 2014 Jun 22;14:165. doi: 10.1186/1471-2180-14-165 (PMC4076439; doi:10.1186/1471-2180-14-165)
Supplement: Additional file 2: Table S2 — Genetic characterization of resistance genes and MIC values for amikacin, kanamycin and capreomycin in 27 AK- and KM-susceptible clinical isolates of M. tuberculosis. [file 1471-2180-14-165-S2.doc]

**Additional file 2: Table S2** Genetic characterization of resistance genes and MIC values for amikacin, kanamycin and capreomycin in 27 AK- and KM-susceptible clinical isolates of *M. tuberculosis*

| **Isolate** | **MIC (µg/ml)** | | |  | ***rrs* mutation** |  | ***eis* promoter mutation** | ***tap* mutation** | | ***whiB7* promoter mutation** | ***tlyA* mutation** | |
| --- | --- | --- | --- | --- | --- | --- | --- | --- | --- | --- | --- | --- |
|  | **AK** | **KM** | **CAP** |  |  |  |  | **Nucleotide change** | **Amino acid change** |  | **Nucleotide change** | **Amino acid change** |
| DS004 | 2-4 | 4 | 2-4 |  | wt |  | wt | wt | wt | wt | A33G | wt |
| DS016 | 2-4 | 4 | 2-4 |  | wt |  | wt | wt | wt | wt | A33G | wt |
| DS039 | 2-4 | 4 | 2-4 |  | wt |  | wt | Ins581C | Frameshift (419>231) | wt | A33G | wt |
| DS165 | 2-4 | 4 | 2-4 |  | wt |  | wt | Ins581C | Frameshift (419>231) | wt | A33G | wt |
| DS280 | 2-4 | 4 | 2-4 |  | wt |  | wt | wt | wt | wt | A33G | wt |
| DS305 | 2-4 | 4 | 2-4 |  | wt |  | wt | wt | wt | wt | A33G | wt |
| DS307 | 2-4 | 4 | 2-4 |  | wt |  | wt | wt | wt | wt | A33G | wt |
| DS320 | 2-4 | 4 | 2-4 |  | wt |  | wt | wt | wt | wt | A33G | wt |
| DS378 | 2-4 | 4 | 2-4 |  | wt |  | wt | wt | wt | wt | A33G | wt |
| DS379 | 2-4 | 4 | 2-4 |  | wt |  | wt | wt | wt | wt | A33G | wt |
| DS386 | 2-4 | 4 | 2-4 |  | wt |  | wt | wt | wt | wt | A33G | wt |
| DS394 | 2-4 | 4 | 2-4 |  | wt |  | wt | wt | wt | wt | A33G | wt |
| DS563 | 2-4 | 4 | 2-4 |  | wt |  | wt | wt | wt | wt | A33G | wt |
| DS596 | 2-4 | 4 | 2-4 |  | wt |  | wt | Ins581C | Frameshift (419>231) | wt | A33G | wt |
| DS644 | 2-4 | 4 | 2-4 |  | wt |  | wt | wt | wt | wt | A33G | wt |
| DS684 | 2-4 | 4 | 2-4 |  | wt |  | wt | wt | wt | wt | A33G | wt |
| DS706 | 2-4 | 4 | 2-4 |  | wt |  | wt | wt | wt | wt | A33G | wt |
| DS722 | 2-4 | 4 | 2-4 |  | wt |  | wt | Ins581C | Frameshift (419>231) | wt | A33G | wt |
| DS736 | 2-4 | 4 | 2-4 |  | wt |  | wt | wt | wt | wt | A33G | wt |
| DS747 | 2-4 | 4 | 2-4 |  | wt |  | wt | Ins581C | Frameshift (419>231) | wt | A33G | wt |
| DS812 | 2-4 | 4 | 2-4 |  | wt |  | wt | wt | wt | wt | A33G | wt |
| DS851 | 2 | 4 | 4 |  | wt |  | wt | wt | wt | wt | A33G | wt |
| DS859 | 2-4 | 4 | 2-4 |  | wt |  | wt | wt | wt | wt | A33G | wt |
| DS874 | 2-4 | 4 | 2-4 |  | wt |  | wt | wt | wt | wt | A33G | wt |
| DS877 | 2-4 | 4 | 2-4 |  | wt |  | wt | wt | wt | wt | A33G | wt |
| DS945 | 2-4 | 4 | 2-4 |  | wt |  | wt | wt | wt | wt | A33G | wt |
| DS1945 | 2-4 | 4 | 2 |  | wt |  | wt | wt | wt | wt | A33G | wt |
| H37Rv | 2 | 4 | 4 |  | wt |  | wt | wt | wt | wt | wt | wt |

wt, wild-type; Ins581C, Insertion of cytosine at position 581; A33G, Nucleotide change at position 33 from adenine to guanine; Frameshift (419>231), Mutation caused size reduction from 419 to 231 residues
